# Functional Alterations in Resting-State Visual Networks in High-Tension Glaucoma: An Independent Component Analysis

**DOI:** 10.3389/fnhum.2020.00330

**Published:** 2020-08-12

**Authors:** Yi Wang, Weizhao Lu, Yuanzhong Xie, Jian Zhou, Tingqin Yan, Wenhui Han, Jianfeng Qiu

**Affiliations:** ^1^Department of Ophthalmology, The Second Affiliated Hospital of Shandong First Medical University, Taian, China; ^2^Department of Ophthalmology, Shandong First Medical University & Shandong Academy of Medical Sciences, Taian, China; ^3^Medical Engineering and Technology Research Center, Shandong First Medical University & Shandong Academy of Medical Sciences, Taian, China; ^4^Department of Radiology, Shandong First Medical University & Shandong Academy of Medical Sciences, Taian, China; ^5^Department of Radiology, Taian City Central Hospital, Taian, China; ^6^Department of Ophthalmology, Taian City Central Hospital, Taian, China

**Keywords:** glaucoma, intraocular pressure, independent component analysis, visual network, functional MRI

## Abstract

**Background:**

High-tension glaucoma (HTG) is the most common type of primary open angle glaucoma and elevated intraocular pressure (IOP) is the major risk factor of the disease. The aim of this study was to assess alterations in resting-state visual networks in patients with HTG and investigate the effect of elevated IOP on the visual networks.

**Methods:**

T1-weighted and resting-state functional MRI images were acquired from 36 HTG patients (aged 49.22 ± 15.26 years) and 20 healthy controls (aged 49.90 ± 5.62 years). Group independent component analysis (ICA) was utilized to evaluate altered functional connectivity (FC) in resting-state visual networks between HTG patients and healthy controls. Pearson correlation analysis between mean IOP and altered FCs in the visual networks was performed.

**Results:**

ICA demonstrated decreased FCs in HTG group in the left calcarine cortex of the lateral visual network, in the bilateral lingual gyrus of the medial visual network and in the bilateral lingual gyrus of the occipital visual network compared with healthy controls. Furthermore, correlation analysis revealed negative correlation between mean IOP and altered FC within the lateral visual network.

**Conclusion:**

The results suggested reduced FCs between primary and higher visual cortices in HTG brain. The IOP elevation might be responsible for the functional alterations in the visual networks.

## Introduction

Glaucoma is the leading cause of irreversible blindness worldwide (Giorgio et al., 2017). Glaucoma-induced intraocular pressure (IOP) elevation is the major risk factor causing permanent damage to the retinal ganglion cell and optic nerve (Adachi et al., 1996). Based on different levels of IOP, glaucoma can be classified into normal-tension glaucoma and high-tension glaucoma (HTG) using the conventional cutoff IOP of 21 mm Hg (Giorgio et al., 2017).

HTG is one of the most common types of primary open angle glaucoma (POAG) clinically (Giorgio et al., 2017). It is mainly manifested as elevated IOP, accompanied by visual field defects and optic nerve damage (Caprioli and Spaeth, 1986; Adachi et al., 1996). Recently, neuroimaging technologies have demonstrated that HTG patients not only suffer from visual defects, but also have alterations in the central nervous system (CNS) (Frezzotti et al., 2014, 2016; Giorgio et al., 2017). Blood oxygenation level dependent (BOLD) functional magnetic resonance imaging (fMRI), as one of the most popular tools in brain research, has been used to assess brain functional changes in HTG patients (Dai et al., 2014; Huang et al., 2015; Cai et al., 2015; Wuerger et al., 2015; Zhang et al., 2016; Chen W. et al., 2017; Zhou et al., 2017). Several fMRI studies have revealed functional reorganization in the visual cortex and lateral geniculate nucleus in HTG brain (Wuerger et al., 2015; Zhang et al., 2016; Zhou et al., 2017). Functional alterations in the visual pathway and beyond the visual system have also been reported in fMRI studies (Huang et al., 2015; Chen W. et al., 2017). However, the forementioned fMRI studies have evaluated brain functional changes in independent brain regions instead of brain networks. The brain is a complete unity with complex networks connecting different regions. In terms of network scale in HTG brain, an fMRI study has demonstrated network centrality changes in HTG brain (Cai et al., 2015). Altered functional connectivity (FC) in HTG brain has also been assessed by fMRI (Dai et al., 2014; Frezzotti et al., 2016).

Independent component analysis (ICA), a data-driven approach, is widely used for processing and analysis of functional networks in fMRI data (Posner et al., 2014). Without any prior information, this method can effectively determine the functional characteristics of mutually correlated brain components (Posner et al., 2014). Therefore, in the present study, we hypothesized that alterations in the resting-state visual networks would be observed in HTG brain. In addition, these alterations may have some associations with the elevated IOP. In this way, we applied group ICA to extract visual networks and adopt correlation analysis to determine whether there was a relationship between alterations in the visual networks and elevated IOP.

## Materials and Methods

### Subjects

This study was approved by the Ethics Committee of the Shandong First Medical University and was conducted in accordance with the Declaration of Helsinki. Written informed consent was obtained from all the subjects after enrollment. Subjects enrollment was carried out by the affiliated hospital. HTG enrollment criteria were as follows: (1) clinical diagnosis of POAG based on gonioscopy, (2) At least one eye suffered from elevated IOP (IOP ≥ 21 mm Hg), (3) at the early- or intermediate-stage of glaucoma, (4) 40–60 years old with more than 12 years of formal education, (5) right-handed. HTG exclusion criteria were: (1) secondary glaucoma or other ocular disorders that could affect the optic visual pathway, (2) hypertension, impaired glucose tolerance, diabetes, or other metabolic disease according to clinical record, (3) treatment of glaucoma, (4) history of psychiatric or neurological illnesses, (5) MRI contraindications such as active implantable medical devices, ferromagnetic implants, large electrically conductive implants or foreign bodies. The enrollment criteria for healthy controls (HCs) were: (1) 40–60 years old with more than 12 years of formal education, (2) right-handed, (3) both eyes’ IOP fell in the normal range (10 mm Hg < IOP < 21 mm Hg). The exclusion criteria were: (1) Glaucoma or other eye diseases, (2) hypertension, impaired glucose tolerance, diabetes, or other metabolic disease according to clinical record, (3) history of psychiatric or neurological illnesses, (4) MRI contraindications such as active implantable medical devices, ferromagnetic implants, large electrically conductive implants or foreign bodies. Finally, 36 HTG patients (49.22 ± 15.26 years old, 15 males and 21 females) and 20 demographically matched HCs (49.90 ± 5.62 years old, 7 males and 13 females) were enrolled in this study. HTG patients were in the early- and intermediate-stage of glaucoma.

All the enrolled subjects underwent IOP measurement by tonometer. HTG patients underwent a comprehensive ophthalmologic examination including retinal nerve fiber layer (RNFL) thickness measurement and optic disk elevation with optical coherence tomography (Heidelberg Engineering’s Spectralis OCT). However, due to limited conditions, we did not perform cognitive examinations to the enrolled subjects.

### MRI Data Acquisition

A 3.0 T MRI scanner (Siemens Magnetom Skyra 3.0 T) with 32-channel head array coil was used in this research. The enrolled subjects were scanned in a supine, head-first position with cushions on both sides and at the top of the head to reduce motion. Conventional T1WI_MPRAGE sequence was firstly acquired. The scan parameters were as follows: repetition time (TR)/echo time (TE)/inversion time (TI) = 2300/2.29/900 ms, field of view (FOV) = 240 × 240 mm, slice thickness = 1 mm, slice gap = 0 mm, matrix = 256 × 256, number of signal averages (NEX) = 1, flip angle (FA) = 8°, bandwidth = 123.36 Hz, and 176 sagittal slices covering the whole brain. Before resting-state fMRI scan, subjects were instructed to close their eyes and stay awake, calm breathing, keep a clear consciousness and not engaged in any specific thinking activity. To acquire fMRI data, the echo planar imaging (EPI) sequence with following parameters were used: TR/TE = 2000/30 ms, FOV = 240 × 240 mm, matrix = 64 × 64, slices number = 33, slice thickness = 3 mm, slice gap = 1 mm, FA = 90°, scan duration time = 480 s (240 volumes).

### Data Preprocessing

Data Processing and Analysis for Brain Imaging (DPABI^1^) was used for resting-state fMRI data preprocessing. DPABI is based on Statistical Parametric Mapping 12 (SPM12) software and runs on the Matrix Laboratory platform (MATLAB R2014b). The preprocessing steps were as follows: (1) Data format conversion from original DICOM format to NIFTI format; (2) The first 10 volumes of fMRI data were removed; (3) Slice timing; (4) Head motion correction. Head motion thresholds were set at maximum translation < 1.5 mm, maximum head rotation < 1.5 degree and framewise displacement (FD) proposed by Power < 0.2 mm (Yan et al., 2013). All the enrolled subjects passed this threshold and no fMRI data were excluded; (5) The fMRI data were then normalized to the Montreal Neurological Institute (MNI) space using Diffeomorphic Anatomical Registration Through Exponentiated Lie Algebra algorithm; (6) The normalized data were then spatially smoothed with an isotropic Gaussian kernel of 6 mm full−width half−maximum.

### ICA Analysis

The preprocessed fMRI data from the two groups were analyzed together using Group ICA of fMRI Toolbox (GIFT^2^). Group ICA included the following main steps (Lu et al., 2020): (1) Principal components analysis was used to reduce data dimensions; (2) The number of independent components (ICs) was preliminary estimated using the minimal length description (MLD) criterion. Then, the number of ICs for each subject were estimated in the ICASSO software using the Infomax algorithm (repeated 20 times). The number of ICs for all the subjects was selected to be the maximum number estimated across subjects (resulting in 55 ICs); (3) Individual ICs were reconstructed using a dual-regression model to get time courses and spatial maps corresponding to the ICs for each subject; (4) The spatial maps were then transformed to Z scores, indicating the synchronization degree of BOLD signal in each voxel with the mean time course of relevant component.

Three resting-state visual networks were used in this study (Damoiseaux et al., 2006; Laird et al., 2011; Heine et al., 2012), namely, the lateral (or secondary) visual network, the medial (or primary) visual network and the occipital visual network. The lateral visual network consists of the peristriate area, lateral and superior occipital gyrus. The medial visual network encompasses striate and parastriate part. The occipital visual network involved in higher-level visual processing associated with orthography and covert reading, which includes mainly the occipital pole (Laird et al., 2011; Heine et al., 2012). The templates of these three networks were created using wfupickatlas in the SPM12 toolbox.

**FIGURE 1:**
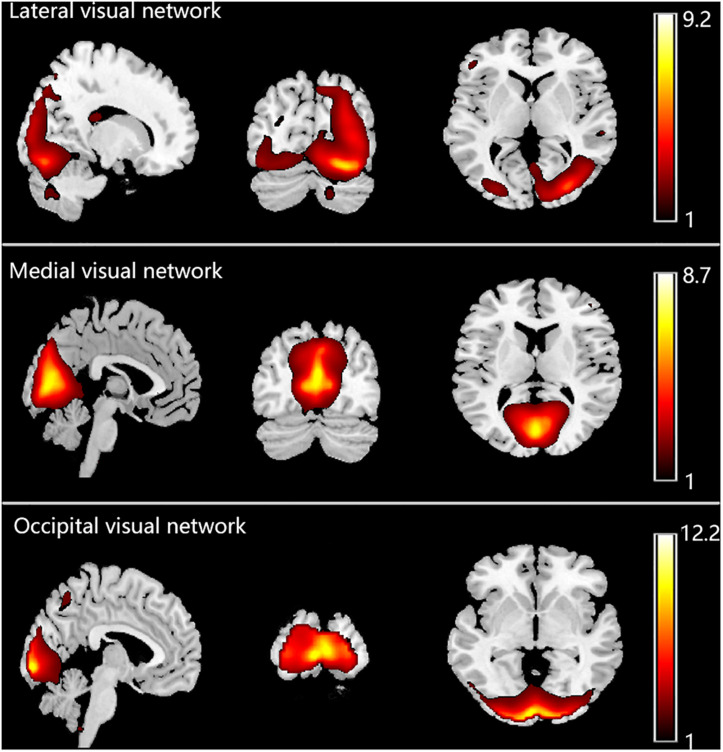
Three visual networks identified in the enrolled 56 subjects.

The ICs were first visually checked to exclude artifacts. To select the three visual networks, spatial correlation analysis was performed for each IC with templates of the three visual networks. Normally, the component with the largest correlation coefficient was selected as the relevant visual network. However, if the IC contained a large portion of white matter or cerebrospinal fluid, the IC with the second largest correlation coefficient was chosen. Finally, three components were identified as the three visual networks we were interested in, as shown in Figure 1.

### Statistical Analysis

Statistical analysis software SPSS 20.0 was used to run independent *t*-test for demographic information and clinical measurements between HTG group and HCs. *P* < 0.05 was considered statistically significant.

The statistical analysis module of DPABI was used to analyze three resting-state visual networks between the two groups. Specifically, spatial maps of the three ICs for all the participants were gathered to generate relevant masks for group analysis using one-sample *t*-test, with a threshold of *p* < 0.05. Then, general linear model was used to detect whether there were significant FC differences within each visual network between HTG group and HCs. Age and gender were treated as nuisance covariates and were entered into the design matrix for linear regression. For multiple comparison correction, family-wise error (FWE) correction was used (two-tailed, *p* < 0.05), and permutation test (10,000 times) with threshold-free cluster enhancement (TFCE) was applied via Permutation Analysis of Linear Models (PALM) approach for cluster-extent based thresholding (Winkler et al., 2016; Chen X. et al., 2017). Minimal cluster threshold of voxels was set at 30, which was close to 1,000 cubic millimeters. The value of Cohen’s d was used to evaluate the effect size (ES).

To explore the effect of elevated IOP on the resting-state visual networks, correlation analysis was performed between regions obtained in the group analysis and the mean IOP in HTG group. Specifically, the regions with significant FC differences in the visual networks were obtained and, for each HTG patient, the mean FC across all voxels of each region was extracted. Pearson correlation analysis was then conducted between the mean FC of these regions and the mean IOP. Bonferroni correction with *p* < 0.05 was considered to indicate statistical significance.

## Results

### Demographic and Clinical Information

Table 1 lists the demographic and clinical information of the HTG group and HCs. The HTG and HC groups were matched by age and gender. Significant differences were found between the two groups in terms of IOP and mean IOP as expected. RNFL and time from diagnosis for HCs were not available.

**TABLE 1:** Demographic and clinical information of high-tension glaucoma patients and healthy controls.

	**HTG**	**HC**	***p*-value**	**T**
Age (years old)	49.22 ± 15.26	49.90 ± 5.62	0.22^*d*^	−0.191
Gender (M / F)	15/21	7/13	0.56^*e*^	NA
IOP_L (mmHg)	35.42 ± 16.16	17.06 ± 3.63	< 0.001^*d*^	4.990
IOP_R (mmHg)	33.94 ± 16.04	15.42 ± 3.68	< 0.001^*d*^	5.069
Mean IOP (mmHg)	34.68 ± 16.01	16.24 ± 1.79	< 0.001^*d*^	6.812
RNFL_L (μm)^*a*^	74.77 ± 33.06	NA^*b*^	NA	NA
RNFL_R (μm)^*a*^	86.25 ± 22.85	NA^*b*^	NA	NA
Time from diagnosis (day)^*c*^	136.39 ± 160.38	NA	NA	NA

**IOP, intraocular pressure; RNFL, retinal nerve fiber layer; NA, not applicable; L, left; R, right; M, male; F, female. ^*a*^Thickness of peripheral retinal nerve fiber layer. ^*b*^The normal range of peripheral retinal nerve fiber layer thickness for healthy subjects is 100 ∼ 120 μm. ^*c*^Time from diagnosis is defined as the time interval between the first diagnosis of HTG and MRI scan. ^*d*^P-value was obtained by independent t-test. ^*e*^P-value was obtained by chi-square test.**

### Group Analysis and Correlation Analysis

**FIGURE 2:**
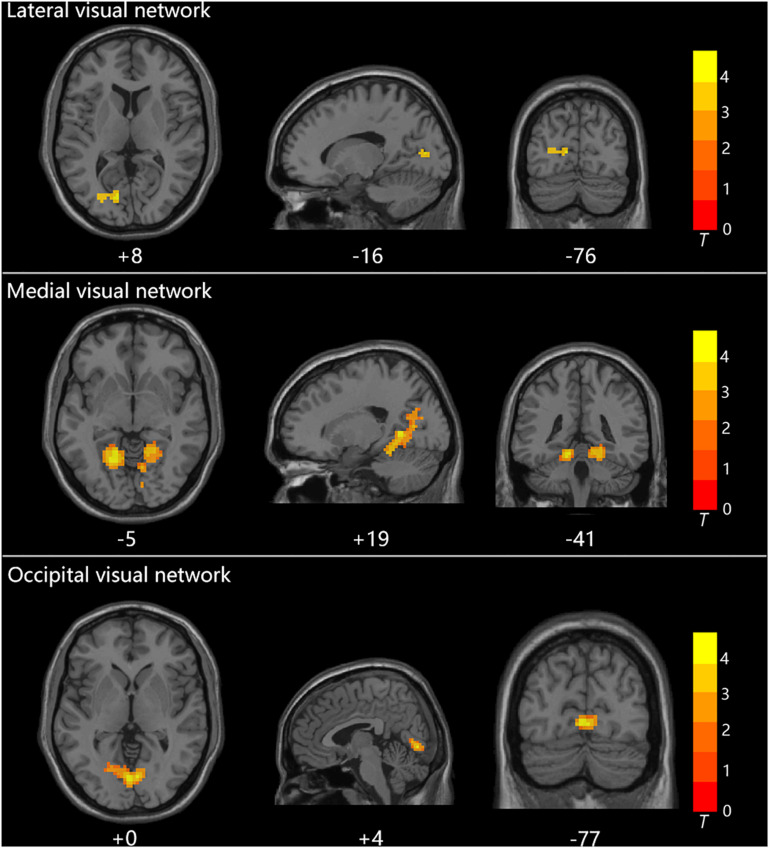
The three visual networks and increased functional connectivities within the networks in healthy controls as compared with high-tension glaucoma patients.

Group statistical analysis was performed between HTG group and HCs for each resting-state visual network using general linear model. The group analysis results for all three resting-state visual networks are shown in Figure 2. Compared with HCs, HTG patients showed decreased FC in the left calcarine cortex within the lateral visual network (FWE corrected, two tailed, *p* < 0.05, ES = 0.51), decreased FC in the bilateral lingual gyrus (LG) within the medial visual network (FWE corrected, two tailed, *p* < 0.05, ES = 0.55) and decreased FC in the bilateral LG within the occipital visual network (FWE corrected, two tailed, *p* < 0.05, ES = 0.67). The brain regions with altered FCs within the visual networks are provided in Table 2.

**TABLE 2:** Regions exhibited altered functional connectivity within the visual networks in healthy controls as compared to high-tension glaucoma patients.

**Visual networks**	**Region**	**Side**	**Cluster size**	**MNI coordinates^a^**			**T**	**ES**	**Brodmann area**
				***x***	***y***	***z***			
**HCs > HTG**									
Lateral	Calcarine	L	40	−15	−78	9	4.3036	0.51	17,18
Medial	Lingual	B	781	18	−54	0	4.935	0.55	18,19
Occipital	Lingual	B	130	0	−78	0	6.6473	0.67	18,19

**HC, healthy controls; MNI, Montreal Neurological Institute; L, left; B, bilateral; ES, effect size. ^*a*^MNI coordinates represent the coordinates of the voxel with maximal statistical significance in each cluster.**

**FIGURE 3:**
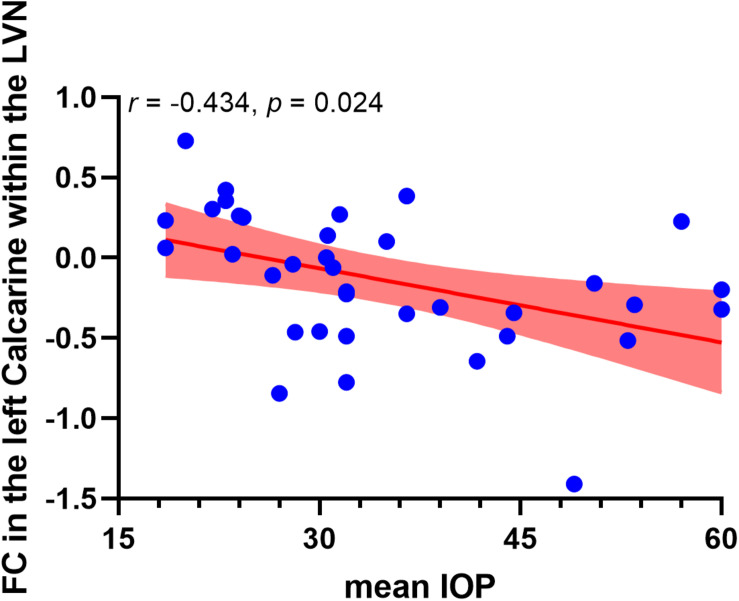
Negative correlation between functional connectivity within the lateral visual network and occipitotemporal visual network and mean IOP in high-tension glaucoma brain. The red area indicates 95% confidence interval. LVN, lateral visual network.

Correlation analysis revealed that the FC in the left calcarine cortex within the lateral visual network showed a negative correlation with the mean IOP (*r* = −0.434, p_*uncorrected*_ = 0.008, p_*corrected*_ = 0.024, Bonferroni corrected). However, altered FCs within the medial visual network and occipital visual network did not show significant correlations with the mean IOP. The correlation analysis result between altered FCs within the visual networks and mean IOP is shown in Figure 3.

## Discussion

In HTG, elevated IOP and other risk factors resulted in the apoptosis of retinal ganglion cells and cross-synaptic degeneration along the visual pathway (Gupta, 2016). The apotosis of retinal ganglion cells and cross-synaptic degeneration along the visual pathway further led to reduced visual information and dysfunction in the central visual cortex (Li et al., 2017).

In the present study, ICA was used to assess FC changes inside the three resting-state visual networks. The results revealed decreased FC within three resting-state visual networks in HTG brain. In HTG group, loss of retinal ganglion cells, visual field damage and degeneration of the visual pathway resulted in reduced visual information to the central visual cortex (Wang et al., 2008). Therefore, the decreased FC within visual cortex and ventral visual pathway may be caused by reduced visual information.

Inside the lateral visual network, decreased FC between the left calcarine cortex and the rest of the visual network was observed. In addition, mean IOP had a negative correlation with the FC within the lateral visual network. The lateral visual network (or the secondary visual network), mainly including Brodmann area (BA) 19, is the lateral part of the visual cortex (Damoiseaux et al., 2006; Laird et al., 2011; Heine et al., 2012). BA 19 is part of higher visual cortex which is considered a visual association area implicated in feature extracting, shape recognition and face perception (Dai et al., 2014; Cai et al., 2015). The calcarine is part of the primary visual cortex which directly receives visual information from visual field, and is involved in basic visual processing (Wang et al., 2008). The decreased FC in the lateral visual network suggested decreased functional connection between primary and higher visual cortices, which may be due to reduced transmission of visual information within the visual cortices caused by elevated IOP. Decreased FC between primary and higher visual cortex was consistent with a previous fMRI study on POAG (Dai et al., 2014). In addition, the results indicated that altered FC within the lateral visual network might be a useful biomarker for investigating IOP elevation and disease progression.

Decreased FC changes were found in the medial visual network. The medial visual network (or the primary visual network), including BA 17 and BA 18, is the primary visual region which receives direct input relating to visual stimuli (Dai et al., 2014; Li et al., 2017). Previous resting-state fMRI studies have found reduced spontaneous neural activities in the primary visual cortices in HTG brain, including decreased amplitude of low-frequency fluctuation (ALFF) values in the LG and cuneus (Li et al., 2015), reduced regional homogeneity (ReHo) values in the middle occipital gyrus, calcarine sulcus and LG (Song et al., 2014; Chen W. et al., 2017). On the network scale, an fMRI study reported significantly decreased degree centrality in the bilateral visual cortices (Cai et al., 2015). FC analysis revealed decreased intrinsic FC between the primary visual cortex and secondary visual cortex (V2) pathway (Li et al., 2017). In line with previous studies, the decreased FC within the medial visual network indicated reduced visual information integration in the primary visual cortex.

In the present study, decreased FC in the bilateral LG within the occipital visual network was observed. The occipital visual network was involved in higher-level visual processing and visual word formation (Laird et al., 2011; Mark et al., 2011). The LG of the occipital gyrus is part of the primary visual cortex related to low-level visual processing, especially related to the identification and recognition of words (Bogousslavsky et al., 1987). In addition, the LG is believed to be involved in encoding visual memories (Roland and Gulyás, 1995). The decreased FC in the bilateral LG within the occipital visual network suggested trans-synaptic degeneration of the visual pathway between the primary visual cortex and higher visual cortex, which was consistent with previous studies (Dai et al., 2014; Wang et al., 2020).

In addition to the decreased FCs in the resting-state visual networks, several MRI studies have reported brain structural alterations both in the visual cortex, visual pathway and non-visual regions of HTG patients (Frezzotti et al., 2014; Chen W. et al., 2017). The decreased FCs in the visual networks bore a strong resemblance to the brain structural alterations. Moreover, several previous fMRI studies also demonstrated increased functional activity in HTG brain, and attributed the phenomena to brain plasticity change or functional compensatory hypothesis in adaption to the disease (Dai et al., 2014; Frezzotti et al., 2014; Song et al., 2014; Li et al., 2015). However, in the present study, no increased FC was found, which made brain plasticity or functional compensatory hypothesis hardly an explanation to our study. The reasons were as follows: (1) The enrolled HTG patients in this study suffered from ultrahigh IOP, which might add difficulty to the visual system and brain in dealing with the disease; (2) The severity and progression of HTG would affect the result of fMRI studies. Previous studies have illustrated that only patients with moderate stages of HTG showed brain plastic change in adaption to the disease (Yu et al., 2015; Frezzotti et al., 2016).

The current concept in the pathophysiology of glaucoma is extended from retinal ganglion cells death to neurodegeneration condition through the entire brain. However, the present ICA only extracts three resting-state visual networks, non-visual system is ignored. In addition, cognitive examinations such as visuospatial and visuoperceptual abilities were not performed due to limited conditions. In statistical analysis, the results may not hold enough statistical power due to relatively small sample size. Future studies should pay more attention to the collection of patients and relevant clinical information.

## Conclusion

In conclusion, this study used ICA to assess FC changes inside resting-state visual networks in HTG brain. The results demonstrated decreased functional connections in the primary visual cortices, between primary visual cortex and higher visual cortex. The FC changes within the visual networks may reflect loss of visual field, reduced visual information to the central visual cortex and decreased connections inside the visual cortices. The hypoconnectivity within the lateral visual network may be related to the elevated IOP.

## Data Availability Statement

The raw data supporting the conclusions of this article will be made available by the authors, without undue reservation, to any qualified researcher.

## Ethics Statement

The studies involving human participants were reviewed and approved by the Ethics Committee of the Shandong First Medical University. The patients/participants provided their written informed consent to participate in this study.

## Author Contributions

YW, YX, JZ, and TY designed and conducted the experiments. WL, WH, and JQ analyzed the data. WL wrote the manuscript. YW, YX, and JQ contributed to the discussion and edited the manuscript. All authors contributed to the article and approved the submitted version.

## Conflict of Interest

The authors declare that the research was conducted in the absence of any commercial or financial relationships that could be construed as a potential conflict of interest.

## Footnotes

1 http://restfmri.net/forum/DPARSF

2 http://mialab.mrn.org/software/
